# Variola Vaccine in Pure Culture

**Published:** 1913-12

**Authors:** W. Fornet

**Affiliations:** Berliner Klinische Wochenschrift


### SPECIAL ABSTRACT

### Variola Vaccine In Pure Culture

Staff Physician Dr. W. Fornet, *Berliner Klinische Wochenschrift,* October 6, 1913.

Editorial Note.—News of this discovery reached us in time for the November issue, but the interest of the subject and recent experience of others in accepting too readily similar announcements, made is advisable to consult with Dr. C. A. Ewald, our associate in Germany, and to await the receipt of the full report. Dr. Ewald endorses Dr. Fornet's work as trustworthy. The *Wochenschrift* did not arrive in Buffalo until October 29, when most of the November issue had been printed.

After citing the well known limitations to the permanence of glycerinated lymph and describing preliminary experiments, the author describes his method in detail. Fifty centigrams of raw lymph without addition of glycerin or several small pox pustules are poured with 20 c.c. of ether into a 50 c.c. powder bottle. The glass stopper is secured with string and a rubber cap and agitated by machinery for twenty-four hours at room temperature. Cultures are taken in bouillon, agar on the slant and deep. If necessary, the process is continued till the material is shown to be sterile, under both ærobic and anærobic conditions. This furnishes a very active vaccine to which the specific culture methods can be further applied. For this purpose, Pasteur glass cylinders serve best. Wadding is placed in the glass cap which is closed by a valve, such as are sold for the inner tubes of automobile tires. The cylinder is two-thirds filled with cattle serum or ascites bouillon (1/3 serum plus 2/3 sugar bouillon), a bit of spongy platinum being added to facilitate anærobiosis. The inoculated and well closed cylinder is now placed in an anærobic apparatus from which the air has been exhausted as far as possible with a filter pump. At this time the automobile valve is opened, while it is again closed during the following introduction of hydrogen. Repeated making and breaking of the vacuum, incubation at 37 degrees C. for 5-10 days, follow. Reinoculation on fresh tubes of serum bouillon, and preservation of the inoculated tubes in the ice box. When a considerable number of cultures and daughter cultures have been secured, the testing on the calf or on children follows. Smears are also made and are stained with Löffler or Giemsa solution for twenty-four hours, or are examined with the dark field.

The author alludes to the analogy of other filterable viruses, such as that of rabies and of poliomyelitis, recently described by Flexner and Noguchi. He refers to the organism as the Microsoma variolæ *seu* vaccinæ and plainly implies an identity of the two. (Note—While this theory is an old one, the minority view for a good many years, probably the majority one recently, it has usually been conceded, even by those who did not believe small pox and cow pox to be essentially different diseases, that the virus of vaccina was essentially and permanently mitigated by passing through the calf and some have even given a complicated, illustrated explanation of shortening of the life cycles of the germ. Certain strains of bovine vaccine have been historically traced back to the inoculation of the first calf with human, small pox virus, but, so far as we know, this is the first time that a vaccine has been directly prepared from variolar lesions. It does not seem that "einige Pusteln von Pockenkranken" can mean anything else. It is stated also that these cultures can be secured in the same way from genuine small pox as from cow pox, and no explicit warning is given nor any statement that the cultures derived from these two sources are to be differently used or give different degrees of reaction. This matter is of so much importance—possibly even medico-legally, since the old laws against inoculation may still be in force—that we have made these statements as clearly as possible and invite criticism.)

Macroscopically, the inoculated cultures scarcely differ from uninoculated tubes, except that after long incubation a slight cloudiness develops, especially near the spongy platinum. Microscopically, nothing was found in fresh hanging drop and stained preparations. Only after the application of hot carbol-fuchsin, strong Giemsa solution and a mordant, especially Löffler's, were very small, round corpuscles visible, 0.2-0.5 microns in diameter. In their characteristic arrangement, they lay in pairs, surrounded by a slight halo and connected by a delicate bridge. Magnified 2000 times and viewed in the dark field, a special differentiation of the center was noted. The question as to whether these were really minute organisms or precipitates of albumin or from stains suggested itself since Huntemüller had found quite similar appearances in uninoculated cultures. But the complete rounding of the forms, the dominant arrangement by pairs and the unanimous agreement of stained and unstained dark-field specimens, lead to the opinion that they are really micro-organisms. Their resistance to disinfection of various kinds and biologic peculiarities exclude them from the bacteria.

Similar forms have been noted by Karl Weigert, 1874; Renault, 1881; Pohl-Pincus, 1882; Gorini, 1900; Calmette and Guerin, 1901; Dombrowski, 1902; Bosc, 1904; Prowazek, 1905; Paschen, 1906; Casagrandi, 1906; Volpino, 1907; and others in smears and sections. Robert Koch, 1881, found similar bodies in sections of the kidney of a small pox patient, with a magnification of only 700.

The author concludes: that raw lymph can be freed from extraneous germs by shaking with ether, twenty hours usually suffice, but no damage is done by extending the period to 150 hours; the sterilized lymph is as efficient as glycerinated lymph, which is not absolutely sterile; the sterile, ether-lymph is stable for a long time, even at rather high temperatures; the virus can be inoculated from one tube to another but loses somewhat in virulence; cultures can be secured either from genuine small pox or from cow pox; the microsoma variolæ seu vaccinæ (note that he uses the conjunction seu and not aut) is the active germ, since it is the only living organism in the active cultures; it resembles in the minutest details the various descriptions of many previous authors.

